# Psychological stressors affecting midwifery students in clinical education: a systematic review

**DOI:** 10.1186/s12909-025-08051-4

**Published:** 2025-10-21

**Authors:** Robabe Seyedi, Sara Dousti, Fatemeh Shabani, Sepideh Hajian

**Affiliations:** 1 Student Research Committee, School of Nursing and Midwifery, Shahid Beheshti University of Medical Sciences, Tehran, Iran; 2 Department of Midwifery, School of Nursing & Midwifery, Shahid Beheshti University of Medical Sciences, Tehran, Iran

**Keywords:** Midwifery, Education, Psychological, Stress, Student

## Abstract

**Background:**

Midwives play a significant role in reducing maternal and neonatal morbidity and mortality. As such, providing high-quality, low-stress clinical training is crucial to the professional development of midwifery students. Identification and resolution of psychological stressors are crucial to improve training programs and, in turn, maternal and neonatal health outcomes. The current study examines the psychological stressors experienced by midwifery students during their clinical training.

**Materials and methods:**

This systematic review included 11 cross-sectional studies selected using the PRISMA checklist. Relevant literature was retrieved from Medline, Embase, CINAHL, Scopus, Web of Science, and Google Scholar databases, covering publications from 1990 to 2024. Keywords such as"midwifery," "education," "psychological stress," and "student," along with their MeSH equivalents, were used. Study selection and data extraction were conducted independently by two researchers. The methodological quality of the included studies was assessed using the Newcastle-Ottawa Scale.

**Results:**

The main categories of stress-related factors in midwifery clinical education identified in this review were: (1) Interpersonal Challenges and Inadequate Student Support; (2) Environmental and individual stressors; (3) Structural and educational challenges; and (4) The mediating role of personal and personality traits in clinical stress.

**Conclusion:**

Psychological stress experienced by midwifery students in clinical settings is a significant challenge. This stress arises from humiliating interactions, lack of support, gaps in education and structure, as well as environmental and individual stressors. Identifying these stressors is crucial for developing targeted interventions that can enhance students' clinical learning and ultimately improve midwifery education.

## Background

According to the International Confederation of Midwives (ICM), midwives play a key role in providing primary care during pregnancy, normal childbirth, and the postpartum period [1]. Therefore, providing high-quality clinical training opportunities is essential for educating students in health sciences disciplines, including midwifery [2]. To become competent practitioners, midwifery students require in-depth theoretical knowledge and extensive practical skills. They must enhance their practical skills through hands-on experience and practice in real clinical environments [3]. During this process, students must learn complex concepts and face clinical and practical challenges, which can serve as stressors and negatively affect their quality of life [4, 5].

Psychological stress is a physiological and psychological response to internal and external demands that disrupt an individual’s equilibrium. While moderate stress can enhance learning performance, excessive or chronic stress can impair cognitive function, disrupt the learning process, and negatively affect overall student health [6–8]. Clinical training, while essential for the development of professional knowledge, skills, and competencies, is also one of the most significant sources of stress for students [9].

Previous research in medical sciences shows that students experience significant stress during clinical training. In nursing, patient care and interactions with teaching and clinical staff are primary stressors, managed predominantly through problem-solving behaviors [10]. Among medical students, low resilience and exposure to challenging clinical events increase stress and negatively affect mental health and academic performance, whereas resilience-focused programs can mitigate these effects [11]. These findings highlight that clinical stress and coping strategies are critical across disciplines.

Studies indicate that midwifery students experience high levels of stress during clinical training, negatively affecting learning quality, clinical performance, and mental and physical health [12–14]. Stressors include clinical workload, challenging interactions with instructors and staff, fear of clinical errors, and difficulties transitioning to professional roles [15–17]. Despite its significance, existing evidence is fragmented and lacks systematic synthesis. Consolidating data on stress sources can inform educational programs to integrate effective coping strategies, ultimately fostering competent and resilient midwives [14]. Therefore, a comprehensive systematic review of psychological stressors in midwifery clinical education is essential to unify current evidence and guide interventions to enhance training quality.

## Methods

This systematic review was conducted to investigate the stress-inducing factors in the clinical education environment of midwifery students, following the Preferred Reporting Items for Systematic Reviews and Meta-Analyses (PRISMA) checklist for systematic reviews. It should be noted that all the included studies were cross-sectional in design.

### Search strategy

A comprehensive literature search was conducted in electronic databases including Google Scholar, Medline, Embase, CINAHL, Scopus, and Web of Science. The search strategy incorporated keywords such as “midwifery”, “education”, “psychological stress”, and “student”, along with their MeSH equivalents. The search covered studies published between June 26, 1990, and June 26, 2024. Both English and Persian language articles were included (Details of the search strategy are presented in Table 1).

**Table 1:** Search strategy

Ovid MEDLINE	
1	(exp Midwifery/ and exp Students/) or (midwi* adj3 (student* or learner* or trainee*)).ti,ab,kf.
2	exp Midwifery/ed or (exp Midwifery/ and exp Education/) or (midwi* adj3 (educat* or train* or teach*)).ti,ab,kf.
3	exp Stress, Psychological/ or stress*.ti,ab,kf.
4	1 or 2
5	3 and 4
Embase	
#1	'midwifery student'/exp OR (midwife/exp AND student/exp) OR (midwi* NEAR/ NEAR/3 (student* OR learner* OR trainee*)): ti,ab,kw
#2	'midwifery education'/exp OR (midwife/exp AND education/exp) OR (midwi* NEAR/3 (educat* OR train* OR teach*)): ti,ab,kw
#3	'mental stress'/exp OR stress*:ti,ab,kw
#4	#1 OR #2
#5	#3 AND #4
#6	#5 NOT 'conference abstract'/it
CINAHL (EBSCOhost)	
S1	(MH "Students, Midwifery+") OR ((MH Midwifery+) AND (MH Students+)) OR (midwi* N3 (student* OR learner* OR trainee*))
S2	(MH "Education, Midwifery+") OR ((MH Midwifery+) AND (MH Education+)) OR (midwi* N3 (educat* OR train* OR teach*))
S3	(MH "Stress, Psychological+") OR stress*
S4	S1 OR S2
S5	S3 AND S4
Scopus	
#1	TITLE-ABS-KEY(midwi* W/3 (student* OR learner* OR trainee*))
#2	TITLE-ABS-KEY(midwi* W/3 (educat* OR train* OR teach*))
#3	TITLE-ABS-KEY(stress*)
#4	#1 OR #2
#5	#3 AND #4
Web of Science Core Collection (SCIE, SSCI, and ESCI)	
#1	TS=(midwi* NEAR/3 (student* OR learner* OR trainee*))
#2	TS=(midwi* NEAR/3 (educat* OR train* OR teach*))
#3	TS=stress*
#4	#1 OR #2
#5	#3 AND #4

## Inclusion and exclusion criteria

This review included observational (cross-sectional) studies that investigated stressors in the clinical learning environment of midwifery students. Studies that focused on non-midwifery students, other educational domains, or employed qualitative designs were excluded from the analysis, because including studies with different methodologies could limit the comparability and synthesis of quantitative results. Additionally, articles were excluded if the full text was not available, were published in languages other than Persian or English, or lacked extractable results and data.

## Study selection process

All retrieved records were imported into EndNote version 21, and duplicate articles were removed. Two researchers independently screened the titles and abstracts, and articles potentially relevant to the topic were selected for full-text review. Subsequently, two reviewers independently assessed the full texts against the inclusion criteria.

In the next phase, data from each study were extracted in a structured format, including authors’ names, year of publication, country, study design, sample size, and main findings. This process was conducted independently by two reviewers to ensure accuracy and completeness of the results. Any discrepancies regarding study inclusion or data extraction were resolved through discussion with the research team. If consensus was not achieved, a third reviewer made the final decision.

**Fig. 1:**
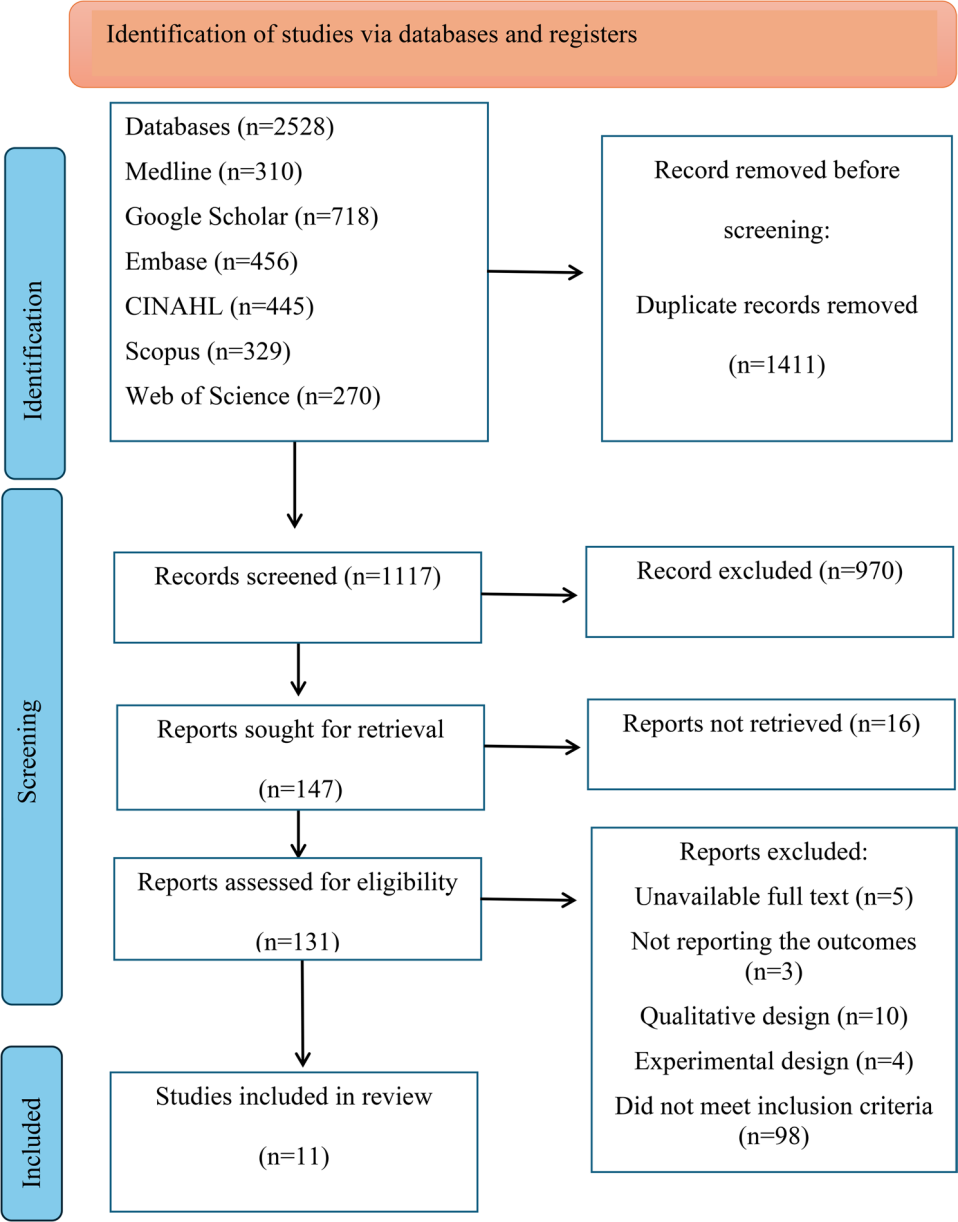
The PRISMA flow diagram of the study

The study selection process is presented in a PRISMA-compliant flowchart (Fig. 1), providing a transparent overview of the systematic review procedure.

## Quality assessment

The methodological quality of the included studies was independently assessed by two reviewers using the Newcastle-Ottawa Scale (NOS) for cross-sectional studies [18]. Discrepancies were resolved through unanimity. Out of the total studies, five (45%) were rated as high quality (scores 7–9), three (27%) as moderate quality (scores 4–6), and three (27%) as low quality (scores ≤ 3) (Table 2). No study was excluded from the final analysis due to high risk of bias or low quality.

**Table 2:** Quality assessment of the included studies

Author	Year	Selection				Comparability		Outcome		
		Representativeness	Sample size	Non-respondents	Ascertainment of the screening tool	confounding factors	Assessment of outcome		Statistical test	Total Score
Cavanagh	1997	1	0	1	2	1	2		1	8
Poorheidari	2018	1	0	0	1	0	1		0	3
Jajvandian	2022	1	0	1	1	0	2		1	6
Moridi	2010	1	1	0	1	0	2		1	6
Rezaei	2020	1	0	1	2	1	2		1	8
Khajehei	2011	0	0	0	1	0	1		0	2
Budu	2019	1	1	0	2	1	2		1	8
Seyedamini	2018	1	1	1	1	1	2		1	8
Cilingir	2011	1	1	1	1	1	2		1	8
Ziaee	2014	1	1	0	1	1	2		1	7
Yazdizadeh	2016	0	1	0	1	0	1		0	3

## Results

This systematic review was registered in the International Prospective Register of Systematic Reviews (PROSPERO) under the ID: CRD42024561244. A total of 2,528 records were retrieved from six databases. After removing 1,411 duplicates, 1,117 records remained for screening. Following the screening process, 11 studies comprising a total of 1,607 participants were included in the final review. A summary of the included studies is presented in Table 3.

**Table 3:** Summary of the reviewed studies

Authors	Year	Country	Aim	Design	Study population	Research instrument	Key finding
Cavanagh	1997	England	Educational sources of stress among student midwives working in England.	Descriptive-Analytical	127 preregistration and 74 shortened preregistration responses from midwifery students	Researcher-developed questionnaire	stress associated with the organization of the learning experience: Insufficient time to complete assignments (22.89%), Receiving incomplete information about assigned tasks (27.87%), Working hard but receiving low grades (34.33%), Having too little time to meet expectations (20.4%), Placement staff being unclear about the student’s role (18.41%), Being singled out for criticism while others are not (22.89%), Insufficient preparation for examinations (19.41%) Sources of home and family stress: Managing conflicts between domestic responsibilities and personal demands (20.4%), Concerns regarding securing employment after completing the course (44.48%), Exposure to ‘life and death’ situations during placements (21.9%)
Poorheidari	2018	Iran	to investigate the stressful experiences of midwifery students during clinical education in the labor room.	Descriptive-Cross-Sectional	71 midwifery students who had internship or apprenticeship experience	Researcher-developed questionnaire	unpleasant emotions (3.2 ± 0.75), instructor training method (3.01 ± 0.79), clinical experience (2.94 ± 0.86), educational planning (2.86 ± 0.65), clinical education environment (2.63 ± 0.7) and interpersonal communication(2.06 ± 0.74) respectively, had higher levels of stress.
Jajvandian	2022	Iran	Determining the stress factors in the clinical education of midwifery students.	Cross-Sectional goal-oriented study	85 midwifery students in North Khorasan province	Personal information questionnaire and a survey questionnaire on stress factors in clinical education, including stress factors in eight areas	The areas of unpleasant feelings and clinical experiences, patient education environment, and students’ skills were ranked as low-stress, with respective scores of 196, 145, 174, and 171. Clinical instructor and clinical experiences areas with scores of 225 and 254 were ranked as medium stress. The student’s personality was ranked as stress-free with a score of 132.
Moridi	2010	Iran	Stressors of clinical education from the perspective of nursing, midwifery, and operating room students.	Descriptive study	230 students of nursing, midwifery, and operating room who had passed at least one clinical course	The Researcher made a questionnaire	In the interpersonal domain, “the manner of instructor behavior toward students” with a mean ± standard deviation of 4.27 ± 0.78; In the humiliating experiences domain, “being reprimanded by instructors in the presence of ward staff, physicians, patients, and their companions” with a mean ± standard deviation of 3.98 ± 1.09; In the educational environment domain, “insufficient attractiveness of the clinical ward” with a mean ± standard deviation of 3.78 ± 0.84; In the clinical experiences domain, “lack of skills in patient care” with a mean ± standard deviation of 4.02 ± 0.80; And in the unpleasant feelings domain, “fear of lacking knowledge and skills” with a mean ± standard deviation of 4.00 ± 0.76.
Rezaei	2020	Iran	This study aimed to explore (1) the perceived stress and stressors of midwifery students and (2) the relationships between students’ stress and related factors in the clinical learning environment.	A Cross-Sectional survey design	108 students were selected using Krejcie and Morgan table in 2016	The Persian version of Cohen’s Perceived Stress Scale (PSS) and the Persian Questionnaire of Stressful Sources (PQSS) in clinical learning	The highest stressors were included: “feeling suffering due to seeing patients with critical situations”, “instructor’s admonition in the presence of clinical staff”, and “communication with instructor”. The “interest in the field of study” hurt perception of stressors in dimensions of “clinical practices” and “interpersonal communication”.
Khajehei	2011	Iran	To investigate various sources of stress among midwifery students.	A Cross-Sectional design	90 midwifery students	The Researcher made a questionnaire	Relationship with Clinical Instructors: 72.2% of third-year students and 50% of final-year students reported that their mistakes led to adverse reactions from instructors and a reduction in their grades. Clinical Environment and Staff Behavior: Sixty-six and two-thirds percent of third-year students and fifty-two and eight% of final-year students stated that hospital staff did not support them and reported their mistakes to the instructors. Nursing Activities: 40.74% of third-year students and 15.55% of final-year students indicated that activities such as changing bed linens and administering injections, fear of medical errors, continuous supervision, and high expectations were sources of their stress.
Budu	2019	Ghana	This study assesses the genesis of stress among midwifery students in Ghana and its impact on their academic performance.	Cross-Sectional design	160 midwifery students	Data was drawn from a 17-item modified response from the College Undergraduate Stress Scale (CUSS)	A significant proportion of students reported experiencing notable stress, with 94.4% of third-year students and 91.7% of final-year students indicating high levels of stress. Primary Sources of Stress: Key stressors identified included: Relationships with clinical instructors (reported by 72.2% of third-year and 50% of final-year students)و Clinical environment and staff behavior (reported by 66.7% of third-year and 52.8% of final-year students)و Nursing activities such as bed-making, administering injections, fear of medical errors, constant supervision, and high expectations (reported by 40.7% of third-year and 15.6% of final-year students).
Seyedamini	2018	Iran	The Effective Stressors in Clinical Education for Nursing and Midwifery Students.	Cross-Sectional study	180 nurses and 120 midwifery students	The Researcher-made a questionnaire	Midwifery students experienced the highest level of stress in the domain of environmental factors, with a mean score of 3.71 ± 0.57, primarily attributed to overcrowding of fellow students in hospital wards (mean: 4.39 ± 0.89). In the domain of educational planning, the average stress level was reported as 3.58 ± 0.70, with the shortage of clinical training hours identified as the primary contributing factor (mean: 3.83 ± 1.31). Regarding unpleasant experiences, the fear of contracting infectious diseases (such as HIV and hepatitis) emerged as the most significant stressor (mean: 3.54 ± 1.19). Additionally, a heavy academic workload (mean: 4.03 ± 1.11) and concerns about future employment (mean: 3.85 ± 1.17) were identified as significant sources of stress within the domains of academic and personal–social factors.
Cilingir	2011	Turkey	Define the expectations that nursing and midwifery college students have of their educators, as well as the stressors they perceive during their education.	Descriptive study	Three hundred forty-five were from the nursing department (96.6%) and 129 came from the midwifery department (92.1%)	The researchers developed a questionnaire based on the existing literature	Instructors’ questions about patients (24.6%), Fear of failure and making clinical errors (16%), Constant presence and direct supervision by instructors in the clinical setting (13.2%), Negative behaviors from staff, physicians, or patients (9.8%), and Harsh warnings from instructors during clinical practice (5.8%).
Ziaee	2014	Iran	Determining clinical education stressors from the viewpoint of midwifery students.	Descriptive - Cross-Sectional study	190 midwifery students	The researchers developed a questionnaire	The clinical stress score of students was 2.82 ± 0.62 out of 5. In the view of students, the most crucial stressor area was the educational environment (3.66 ± 0.78), and the most critical stressor factor was the lack of cooperation between staff and service staff with students, as well as differences in healthcare directives (4.1 ± 1.26). The area of interpersonal relationships showed a positive correlation with the three domains of unpleasant feelings, educational planning, and humiliating experience.
Yazdizadeh	2016	Iran	to determine the stressors in clinical education on midwifery students.	Cross-sectional analytic descriptive study	253 midwifery students	The researchers developed a questionnaire	Maximum stressors were in an unpleasant emotion, which is the highest stress factor, and fear of transmission of infectious disease diseases were included as this factor. Comparing tensions between the 5th and 8th semester students showed that the tension level in the 5th semester students was more than the 8th semester students, *p* < 0/001, and the tension In married students, the rate was less than in single students, *p* < 0.036.

The analysis of the selected studies revealed that psychological stress in midwifery students during clinical training is a multidimensional phenomenon. The key themes identified are as follows:

### Interpersonal challenges and inadequate student support

A commonly reported cause of stress, as found in ten different studies, was the negative interactions with instructors, staff, and patients, along with a perceived lack of support in clinical settings, which undermined students’ confidence and reduced their participation in clinical education. Rezaei et al. [15], Moridi et al. [19], and Poorheidari et al. [20] reported that demeaning behaviors—such as public humiliation, ignoring student questions, insulting remarks, and reprimanding in front of patients or staff—were associated with increased anxiety, low self-esteem, and social withdrawal. Similarly, Khajehei et al. [16] and Yazdizadeh et al. [21] emphasized that the lack of support from instructors made students more vulnerable to stressful situations and increased their psychological distress.

Several studies brought to the forefront the importance of positive interactive collaboration between healthcare staff. Seyedamini et al. [22] and Budu et al. [17] found that some clinical staff displayed disrespectful behavior towards students, thus limiting their participation in caring for mothers. Ziaee et al. [23] reported cases of humiliation faced by students from instructors, staff, and even patients, along with a lack of supportive mentorship, leading to increased stress and feelings of isolation. Similarly, Cavanagh et al. [12] noted that a lack of support from instructors and nursing staff, along with unrealistic expectations and unfair criticism, were significant stressors for students.

Moreover, Cilingir et al. [24] concluded that nursing and midwifery students place a high value on receiving understanding and empathy from their teachers, and expect their teachers not to criticize or reprimand them in public. Overall, these findings indicate that teachers’ respectful and supportive behaviors play a significant role in reducing stress and fostering a positive clinical learning environment.

### Environmental and individual stressors

Environmental and individual factors were identified in seven studies as significant sources of stress for midwifery students. Cilingir et al. [24] reported that strict and continuous supervision by instructors, without adequate guidance, increased students’ stress levels. Yazdizadeh et al. [21] also found that the long duration of the specialized training program, the lack of appropriate welfare facilities in clinical settings and transmitting infectious diseases were significant environmental stressors. Jajvandian et al. [25] and Ziaee et al. [23] also reported that inadequate facilities were among the most significant sources of stress in the clinical environment.

Seyedamini et al. [22] reported that students who were both parents and working part-time had faced high levels of stress. In this study, external environmental factors were identified as the most common stressors for midwifery students, primarily due to the large number of students from the same discipline being placed in hospital environments. Cavanagh et al. [12] found that personal life pressures hindered students’ concentration during clinical training, while Budu et al. [17] identified financial hardship and limited access to essential educational resources and clinical tools as major stressors.

### Structural and educational challenges

Educational and structural issues are significant sources of stress for midwifery students, as identified in eight studies. Cavanagh et al. [12] documented that the lack of time to complete assignments, excessive theoretical and clinical workload, inadequate or overly critical feedback from educators, role and responsibility ambiguity, and insufficient educational resources were noteworthy structural and educational issues. Seyedamini et al. [22] identified a lack of internship hours, unclear educational goals, excessive academic workload, and uncertainty about future job prospects as significant causes of stress. Pourheidari et al. [20] and Moridi et al. [19] identified teaching methods, clinical experiences, and educational planning as significant sources of stress. In contrast, Jajvandian et al. [25] highlighted the crucial role of clinical instructors and the quality of clinical experiences.

Rezaei et al. [15] observed that delayed or confusing feedback significantly increased the level of stress, as it restricted students from recognizing and improving their weaknesses. Ziaee et al. [23] indicated that an excessive theoretical and practical workload, a stressful clinical environment, such as labor pain management, and exposure to complicated deliveries, were significant stressors. Yazdizadeh et al. [21] demonstrated that the lengthy duration of the education program, along with the coinciding theoretical and clinical timetables, hurt the quality of learning and the mental health of students.

### The mediating role of personal and personality traits in clinical stress

Ten studies highlighted the significant role of personal and personality traits in mediating clinical stress among midwifery students. Cavanagh [12] reported that fear of clinical errors and exposure to “life-and-death” situations were primary stressors, with limited self-confidence and difficulty balancing commitments further intensifying stress. Pourheidari et al. [20] identified fear of harming oneself or patients and feelings of inadequacy, particularly in caring for high-risk patients, as key individual stressors. Similarly, Jajvandian et al. [25] and Budu et al. [17] emphasized that low self-confidence and insufficient clinical skills, especially during complex procedures such as vaginal delivery or neonatal resuscitation, heightened stress.

Moridi et al. [19] and Khajehei et al. [16] highlighted anxiety stemming from a lack of clinical knowledge and skills, as well as fear of medical errors, underscoring the importance of self-efficacy in coping with clinical challenges. Rezaei et al. [15] found that insufficient opportunities for practice, difficulty managing critical patients, and emotional reactions to witnessing patient suffering further exacerbated stress. Seyedamini et al. [22] reported that concern about the transmission of infectious diseases was a significant source of stress. Cilingir et al. [24] demonstrated that fear of making mistakes and bearing responsibility, coupled with personal anxiety in performing sensitive procedures such as neonatal care, were primary stress sources. Finally, Yazdizadeh [21] identified fear of infectious disease transmission as a key individual factor, illustrating how personal concerns can mediate overall clinical stress.

## Discussion

Findings from this systematic review indicate that psychological stressors in the clinical education of midwifery students are multidimensional and deeply rooted in educational, environmental, interpersonal, and individual structures. Despite geographical variations among countries, similar patterns of stress-inducing factors emerged across studies, reflecting the widespread and universal nature of these challenges, with similar stressors reported among nurses and midwifery students in both Eastern and Western cultures [26]. Findings from recent umbrella reviews indicate that prelicensure nursing students experience moderate-to-high stress from academic demands, patient care responsibilities, and interactions with faculty and staff, suggesting that some stressors are common across healthcare disciplines [27]. Similarly, in physical therapy students, the most common stressor was patient care, and the most frequent coping behavior was problem-solving [28]. Furthermore, socio-cultural contexts play a significant role in shaping how stress is experienced; for example, differences in professional expectations, attitudes toward mental health, and levels of social support can influence both the intensity and type of stress across cultures [29].

One of the primary sources of stress for midwifery students is the quality of interpersonal relationships and the level of support in the clinical environment [15, 19, 20]. This issue stems from hierarchical educational structures in which students occupy lower power positions and have limited ability to express their needs [23]. Psychological and educational dependence on clinical instructors, especially during the early stages of learning, further exacerbates this vulnerability [30, 31]. The absence of structured support and constructive interaction from instructors and clinical staff not only impedes learning but also reinforces feelings of rejection and anxiety [13]. In fact, instructors’ supportive attitudes and behaviors toward students play a more decisive role in reducing stress and enhancing clinical learning than their professional competencies [30]. To mitigate the impact of these challenges and enhance resilience, a multilevel approach is recommended. At the individual level, training in skills such as emotional self-regulation, mindfulness, and peer support can help students manage stress effectively [32]. Transactional Analysis training has been shown to enhance cognitive flexibility and emotion regulation among nursing students, suggesting that similar interventions could be adapted for midwifery students to reduce stress and improve coping in early patient interactions [33]. At the interpersonal and institutional levels, fostering a supportive culture through instructor training in empathy, constructive feedback, and organizational support is crucial for reducing stress and preventing burnout [34].

Lack of clarity in clinical placement objectives and insufficient practical hours can lead to anxiety and feelings of incompetence among students. Providing constructive feedback, clinical simulations, and well-structured educational planning can enhance students’ confidence and performance while reducing academic stress [35, 36]. Structural factors, such as limited study time and concerns about future job demands, also contribute significantly to stress. Teaching time management skills, offering career counseling, and providing short breaks can help alleviate psychological pressure and foster resilience in students [37].

Environmental and individual stressors intensify students’ psychological burden, depriving them of effective practice opportunities and turning learning from a constructive experience into an exhausting one, while simultaneously restricting the development of independent skills [21, 22, 25, 38]. Findings from Kowalska et al. [14] further highlight that financial status and parental support directly shape students’ quality of life and their reliance on adaptive coping strategies. Student resilience to both individual and environmental stressors can be enhanced through two complementary strategies. At a personal level, skills training in time management, problem-solving, coping, and mindfulness exercises improves students’ perception of control and flexibility. At an organizational level, proper staffing, fair allocation of duties, and well-defined learning goals make the learning environment more supportive and enhance students’ general quality of life [4, 32].

Fear of committing errors, performance-related anxiety, and insufficient coping skills were identified as significant stressors. Notably, discipline-specific challenges in midwifery—such as the first exposure to childbirth and neonatal resuscitation—were recognised as the most prominent sources of psychological stress, particularly during the early semesters of training [39]. Uğurlu’s study demonstrated a significant increase in anxiety during students’ initial clinical exposures due to a lack of experience and confidence in managing critical situations [40]. In response to this, the “Midwifery Resilience Model” developed by Hacıköylü has shown promise in reducing perceived stress through the teaching of resilience-building skills [41].

This review has several limitations. First, most of the included studies were conducted in Iran, which may restrict the generalizability of the results to other cultural or educational contexts. However, our analysis did not reveal significant differences across socio-cultural settings. Second, this review excluded grey literature and was limited to articles published in English and Persian, which may have introduced selection bias and reduced the comprehensiveness of the available evidence. Third, the majority of the included studies employed cross-sectional designs and relied on self-reported data, which increases the risk of bias and limits the ability to establish causal relationships. In addition, heterogeneity in the measurement tools and definitions used to assess stress limited the direct comparability of findings across studies. Moreover, most studies focused primarily on identifying stress-inducing factors, while evidence on the effectiveness of stress-reduction interventions was limited.

## Conclusion

This review emphasizes that the psychological stressors that midwifery students experience during clinical education are multifaceted and influenced by multiple educational, environmental, interpersonal, and individual factors. Current research shows that such stressors may negatively affect students’ scholarship, self-esteem, and psychological health. However, the magnitude and nature of such influences may be subject to cultural and situational variations. It may be possible to mitigate the adverse consequences of such stressors by fostering positive relationships among instructors and students, offering well-designed and organized curricula, providing ample clinical resources, and educating students in stress management skills.

## Data Availability

No datasets were generated or analysed during the current study.
